# Fluorescence characteristics of human Barrett tissue specimens grafted on chick chorioallantoic membrane

**DOI:** 10.1007/s10103-015-1839-x

**Published:** 2015-12-04

**Authors:** Jasmin A. Holz, David F. Boerwinkel, Sybren L. Meijer, Mike Visser, Ton G. van Leeuwen, Jacques J. G. H. M. Bergman, Maurice C. G. Aalders

**Affiliations:** Department of Biomedical Engineering and Physics, Academic Medical Center, Meibergdreef 9, 1105AZ Amsterdam, The Netherlands; Department of Gastroenterology and Hepatology, Academic Medical Center, Meibergdreef 9, 1105AZ Amsterdam, The Netherlands; Department of Pathology, Academic Medical Center, Meibergdreef 9, 1105AZ Amsterdam, The Netherlands

**Keywords:** Barrett’s esophagus, Chorioallantoic membrane, Early detection of cancer, Fluorescence spectroscopy, 5-aminolevulinic-acid, Protoporphyrin-IX

## Abstract

To improve (pre)malignant lesion identification in Barrett’s esophagus (BE), recent research focuses on new developments in fluorescence imaging and spectroscopy to enhance tissue contrast. Our aim was to validate the chorioallantoic membrane (CAM) model as a preclinical tool to study the fluorescence characteristics such as autofluorescence and exogenously induced fluorescence of human Barrett’s tissue. Therefore, esophageal biopsy specimens from Barrett’s patients were freshly grafted onto the CAM of fertilized hen’s eggs to simulate the in vivo situation. The BE biopsy specimens stayed between 1 and 9 days on the CAM to study the persistence of vitality. Fluorescence spectroscopy was performed using six excitation wavelengths (369, 395, 400, 405, 410, 416 nm). Obtained autofluorescence spectra were compared with in vivo spectra of an earlier study. Exogenous administration of 5-aminolevulinic-acid to the biopsy specimens was followed by fluorescence spectroscopy at several time points. Afterwards, the biopsy specimens were harvested and histologically evaluated. In total, 128 biopsy specimens obtained from 34 patients were grafted on the CAM. Biopsy specimens which stayed on average 1.7 days on the CAM were still vital. Autofluorescence spectra of the specimens correlated well with in vivo spectra. Administered 5-aminolevulinic-acid to the biopsy specimens showed conversion into protoporphyrin-IX. In conclusion, we showed that grafting freshly collected human BE biopsy specimens on the CAM is feasible. Our results suggest that the CAM model might be used to study the fluorescence behavior of human tissue specimens. Therefore, the CAM model might be a preclinical research tool for new photosensitizers.

## Introduction

Patients with Barrett’s esophagus (BE) are recommended to undergo regular surveillance endoscopy to detect malignant lesions at an early stage. Esophageal adenocarcinoma develops from premalignant stages of dysplasia, which can be histologically graded. These precursor lesions are amenable to curative and minimally invasive endoscopy therapy, due to the low risk of lymph node metastasis. With a low morbidity and mortality compared to esophagectomy and an excellent 5-year survival rate, the timely detection of early dysplasia is of great clinical importance [1–4].

Early changes in tissue during the progression into malignancy occur at a (sub)cellular level and consist of morphological and chemical changes which cannot be seen during standard endoscopic diagnostic procedures such as white light endoscopy. Recent developments to improve real-time diagnostics focus on imaging such as high-resolution white light endoscopy (HR-WLE), confocal endomicroscopy, narrow-band imaging (NBI), and autofluorescence imaging (AFI); the latest suffers from a high false-positive rate [5–11]. Although a lot of effort has been put in these new technologies, none are currently able to clearly discriminate in real time healthy from premalignant tissue during standard surveillance endoscopy. But these (sub)cellular changes in premalignant tissue do affect the optical absorption and scattering properties of the tissue which might be detected by fluorescence spectroscopy [12–15]. In addition, photosensitizers, such as 5-aminolaevulinic acid (5-ALA) may be administered to enhance the tissue contrast [16, 17]. Administration of 5-ALA leads to the formation of the fluorescent protoporphyrin-IX (PpIX). Metabolic differences between tissue types cause concentration variations. At specific times, the concentration of PpIX inside the (pre)malignant tissue [13] is higher compared to normal tissue levels, which shows as increased fluorescence and is therefore often used to improve the discrimination between dysplastic and non-dysplastic epithelium. This so-called photodiagnosis provides real-time information that can be used to red flag areas of interest during medical procedures for the detection of (early) cancer.

For developing new photosensitizers and the characterization of current sensitizers, usually cell experiments and/or animal experiments are used. The physiology and overall drug-cell interactions are far from the in vivo situation. Animal experiments are expensive and cumbersome. A model which is very suitable for studying the localization of the particles and the treatment efficacy is the chick embryo chorioallantoic membrane (CAM) model which uses the well-vascularized chorioallantoic membrane of fertilized chicken eggs on which tumors can be implanted [18–20]. After implantation, angiogenesis allows the tumor to grow. Because of the easy accessibility of the systemic and tumor circulation, it is possible to illuminate the CAM and study the fluorescence yield and, if applicable, treatment effect. The CAM model is the bridge between the preliminary cell experiments and the in vivo tumor-bearing animal models. To mimic the in vivo situation even better, we grafted freshly excised tissue biopsies directly onto the CAM, thereby maintaining the heterogeneity and tumor architecture, which are lost in homogenous cultures of tumor tissue.

Our aim was to validate the CAM model as a preclinical tool to study the fluorescence characteristics of human Barrett’s tissue and therefore the potential use to test future photosensitizers. First, we studied the vitality of freshly human BE tissue biopsies grafted onto the CAM by examining the hematoxylin and eosin (H&E)-stained slices of the harvested biopsies. Furthermore, the autofluorescence spectra obtained from the grafted tissue were compared with in vivo autofluorescence spectra, and the well-known 5-ALA was topically administered to test the CAM model for the usage of photosensitizer. As light source, a multi-wavelength spectroscopy system consisting of several discrete excitation wavelengths around the 405-nm Soret absorption peak of PpIX was used. This setting was chosen to find the optimal excitation wavelength of PpIX in Barrett’s tissue to optimize new imaging and spectroscopy technologies for improved premalignant lesion identification.

## Materials and methods

### Spectroscopy system

A custom-made spectroscopy system was developed (2M Engineering Ltd., Veldhoven, the Netherlands) for endoscopic in vivo measurements comprising a LED with 369 nm (FWHM of 16 nm) and laser diodes at 395, 400, 405, 410, and 416 nm [15]. The system was connected to an optical fiber probe which delivered the excitation light to the tissue and the fluorescence light back to the connected spectrometer USB4000 (Ocean Optics Inc., Dunedin, Florida, USA) and laptop for spectral recording. The distal fiber probe tip had an outer diameter of 2 mm and an inner functional diameter of 0.82 mm which consisted of a bundle of 30 fibers, thus 15 illumination and 15 collection fibers. Proximal, the 15 collection fibers were aligned to the 200-μm entrance slit of the spectrometer. The biopsy specimens were placed perpendicular under the tip of the probe, thus mimicking the in vivo endoscopic setting.

### CAM model

Fertilized hen’s eggs obtained from Drost Loosdrecht BV (Loosdrecht, The Netherlands) were incubated for 3 days in a Polyhatch incubator (Brinsea Products Inc., Titusville, Florida, USA) at 38–39 °C, 60–80 % humidity, rotating every 1 h. At embryonic day 3, a volume of 2–3 mL albumen was removed from the egg using a 21-G needle injected into the air pocket of the egg in order to lower the level of the CAM inside the egg. A window of approximately 1.5 cm^2^ was cut into the outer shell on top of the egg in order to gain access to the CAM, using blades and scissors. After checking if the embryo was alive, the window was covered with transparent plastic and the egg was placed in a hatcher (Brinsea Products Inc., Titusville, Florida, USA) at 37.5 °C, 50–70 % humidity. When outside the hatcher, the eggs were handled on a heating plate at 37 °C in a laminar flow hood.

### Patient selection and biopsy grafting onto the CAM

Patients scheduled for surveillance endoscopy of non-dysplastic BE (NDBE) or work-up or treatment of early Barrett’s neoplasia at the department of Gastroenterology and Hepatology of the Academic Medical Center (AMC) Amsterdam were included. The Medical Ethics Committee of the AMC Amsterdam approved the study and all included patients were informed and signed a consent form. Biopsies of lesions suspicious for dysplasia within the BE and of endoscopically unsuspicious areas of BE were obtained from 34 patients (two to four biopsies per patient). Immediately after the biopsies were obtained, they were transferred into preheated 37.5 °C transport medium (DMEM, FCS, PenStrep, L.Glut, Fungizone).

At embryonic day 6 of incubation, the egg was placed in the laminar flow hood on a heating plate at 37 °C. The plastic cover was lifted from the egg, and the CAM was cleaned at the location for tissue grafting with ethanol-treated lens paper. The freshly obtained biopsy specimens were subsequently spread on a gloved finger and with the help of two pairs of tweezers carefully spread and placed on the CAM. One biopsy specimen was grafted per egg. The transparent cover was closed, and the egg put back in the hatcher.

### Spectroscopy procedure

The egg was placed in the laminar flow hood and positioned under the spectroscopy set-up. All measurements were performed in a dark room. The optical fiber probe was placed about 1 mm above the biopsy specimen, followed by sequential illumination by all light sources and recording of the fluorescence spectra, including a dark measurement (all light sources off). Subsequently, spectra were recorded adjacent to the biopsy specimen and on the CAM only which was approximately 2 cm from the biopsy specimen. Each saved spectrum was composed of the average of three measurements per site. For the fluorescence spectroscopy measurements with 5-ALA administration, 20 μL of 10 mM 5-ALA (Sigma Chemical Co.) dissolved in 0.9 % NaCl solution was topically administered to each biopsy specimen. Fluorescence spectroscopy was performed at several time points between 0 h before and 23 h after 5-ALA administration. After the last measurement, the biopsy specimens were removed from the CAM and fixed in formalin, embedded in paraffin, and cut and stained with hematoxylin and eosin (H&E). Histopathological assessment of the biopsy specimens was performed by an expert GI pathologist. Biopsies which were considered vital were classified into two groups, suspicious for dysplasia called “dysplastic” and not suspicious for dysplasia called “non-dysplastic.” Finally, the embryo was terminated by high-dose isoflurane.

### Data analysis

The obtained autofluorescence spectra of the biopsy specimens on the CAM were analyzed in the same way as the in vivo spectra which we obtained in an earlier study [15]. Pearson’s correlation coefficient *r* and the coefficient of determination *r*^2^ were calculated to assess the relationship between the autofluorescence spectra from the biopsy specimens and the in vivo spectra.

From all emission spectra, obtained from the biopsy specimens used for 5-ALA measurements, the corresponding dark spectrum was subtracted. The intensity ratio *I*_636_/*I*_600_ of the PpIX fluorescence peak at 636 nm to a reference emission wavelength at 600 nm was calculated for each spectrum. This corrects for variations in applied laser power, probe positioning, and system noise. The mean intensity ratios, the standard deviation (SD), and the standard error of the mean (SEM) were calculated. The statistical relevance of differences in intensity ratios per excitation wavelength and time point was determined by repeated measures one-way ANOVA with subsequent Bonferroni correction using Prism 5 (GraphPad Software Inc., La Jolla, California, USA). The results of Bonferroni’s multiple comparison test were considered significant when the *p* value was <0.05.

### Imaging

Images were obtained from the biopsy specimens on the CAM using a Dino-Lite digital microscope in cross-polarized white light imaging mode (AM413ZT, AnMo Electronics Corp., Hsinchu, Taiwan) before and after 5-ALA administration. Fluorescence imaging was performed to obtain an overview of the PpIX distribution on the biopsy specimens and CAM. Fluorescence images were obtained at blue light illumination ranging from 400 to 430 nm (Crime-lite® 2, Foster + Freeman Ltd., Evesham, UK). A digital camera (Nikon D40X) with a long pass filter (GG455, Foster + Freeman) in front of the lens was used to allow only the detection of emitted fluorescence at longer wavelengths than 435 nm.

## Results

### Biopsy assessment and autofluorescence spectra evaluation

**Fig. 1:**
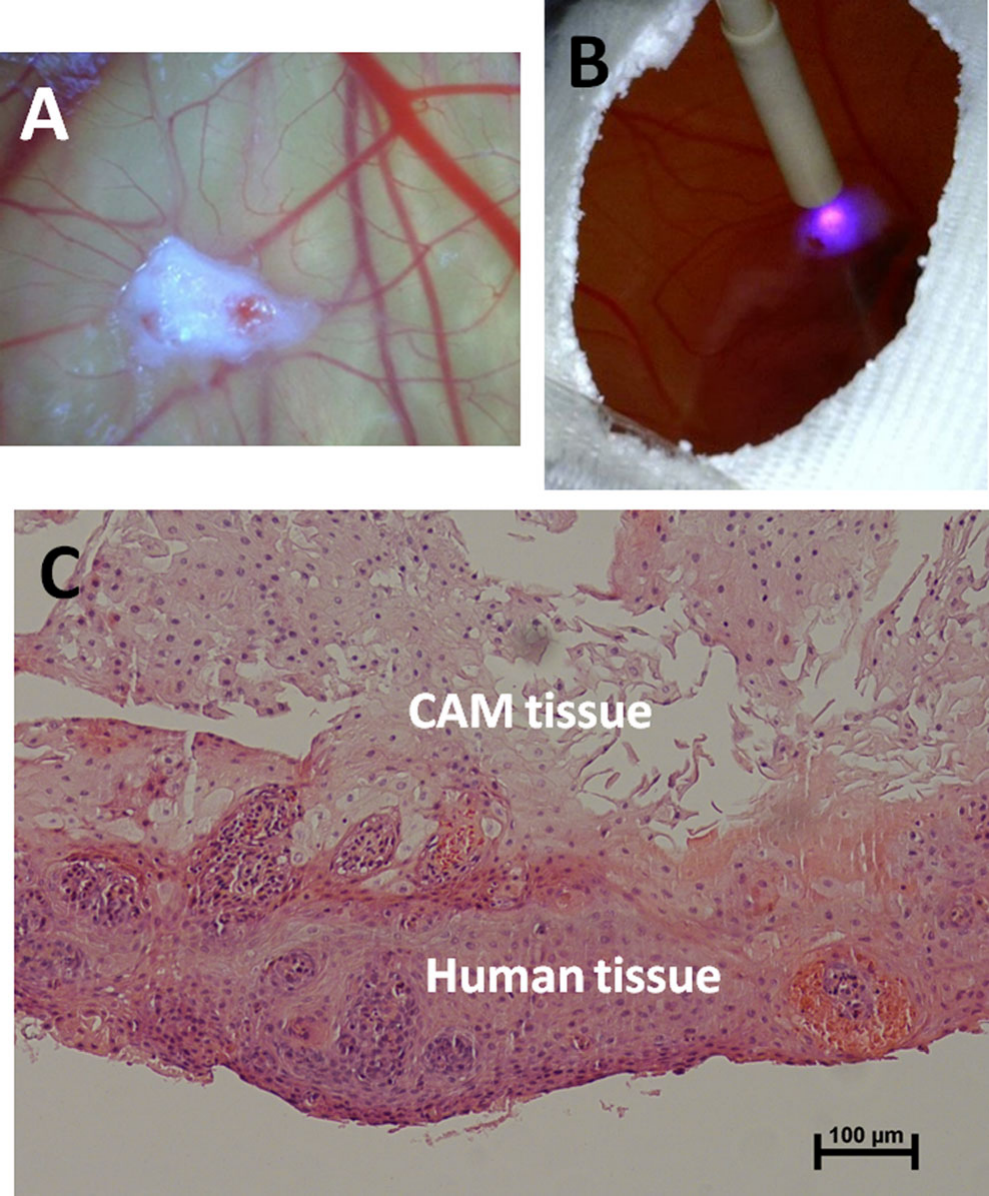
**a** Image of Barrett’s esophageal biopsy specimen on the CAM; **b** fluorescence spectroscopy performed on a biopsy specimen; **c** white light microscope image with ×10 magnification of H&E-stained slice of a human biopsy specimen which stayed 1 day on the CAM

In total, 63 biopsy specimens, obtained from 25 Barrett’s patients, stayed between 1 and 9 days on the CAM. Twenty-six biopsy specimens showed necrosis and were not classified histopathologically. Fourteen other histology slices did not contain enough tissue for an assessment. From the remaining 23 biopsy specimens, 9 were classified as not suspicious for dysplasia (non-dysplastic) thus squamous or non-dysplastic Barrett’s esophagus (NDBE) and 14 as suspicious for dysplasia (dysplastic). The 23 biopsies which could be classified stayed on average 1.7 days on the CAM. Figure 1a shows an image of a Barrett’s biopsy specimen on the CAM.

**Fig. 2:**
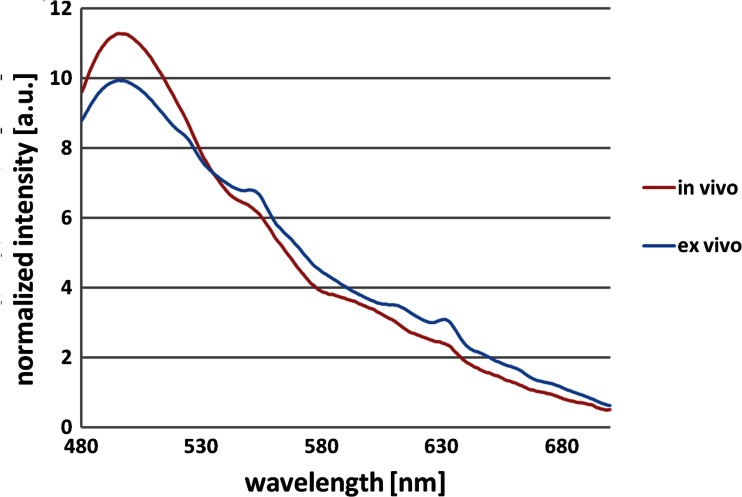
Averaged autofluorescence spectra at 395-nm excitation from in vivo (HGIN/CA) and ex vivo (dysplastic) esophageal tissue

Autofluorescence spectroscopy was performed, see Fig. 1b, on 8 of the 9 non-dysplastic biopsies and on 10 of the 14 dysplastic-classified biopsies. Pearson’s correlation coefficient *r* and the coefficient of determination *r*^2^ (Table 1) were calculated to correlate ex vivo and in vivo averaged autofluorescence spectra obtained with the same system [15]. The ex vivo dysplastic autofluorescence spectra correlated well with the in vivo (HGIN/CA) spectra with an *r*^2^ value of around 0.99. The non-dysplastic spectra correlated less with the in vivo (IM) spectra having an *r*^2^ value of around 0.97. Figure 2 shows the averaged autofluorescence spectra at 395-nm excitation from in vivo (HGIN/CA) and ex vivo (dysplastic) esophageal tissue with an *r*^2^ value of 0.987.

**Table 1:** Coefficient of determination r^2^ of averaged ex vivo versus in vivo autofluorescence spectra for non-dysplastic and dysplastic esophageal tissue

	Non-dysplastic ex vivo vs. in vivo	Dysplastic ex vivo vs. in vivo
369 nm	0.977	0.976
395 nm	0.961	0.987
405 nm	0.961	0.991
410 nm	0.969	0.991
416 nm	0.968	0.991

Figure 1c shows the H&E-stained slice of a human biopsy specimen which stayed 1 day on the CAM. In the upper part, the CAM cells are seen, and in the lower part, the human tissue seemed to be vital and attached to the CAM tissue.

### 5-ALA-induced fluorescence spectra evaluation

A total of 65 biopsies, obtained from 20 Barrett’s patients, stayed between 2 and 4 days on the CAM and were then examined with 5-ALA-induced spectroscopy. Histopathological evaluation of the harvested biopsies was not possible for 25 biopsy specimens due to intensive necrosis and 10 more slices contained not enough tissue for an assessment. From the remaining 30 biopsy specimens, 12 were classified as not suspicious for dysplasia (non-dysplastic) thus squamous or NDBE and 18 as suspicious for dysplasia (dysplastic). Three spectra of non-dysplastic biopsy specimens were excluded from the analysis due to measurement issues. Table 2 gives an overview of the spectra included for the analysis at all six excitation wavelengths obtained from 12 patients.

**Table 2:** Number of analyzed human biopsy specimens on the CAM per time point and their histological classification

Time point	Not suspicious for dysplasia	Suspicious for dysplasia	Sum
0 h	9	18	27
1.5 h	9	18	27
4.5 h	8	16	24
6 h	6	11	17
23 h	2	7	9

**Fig. 3:**
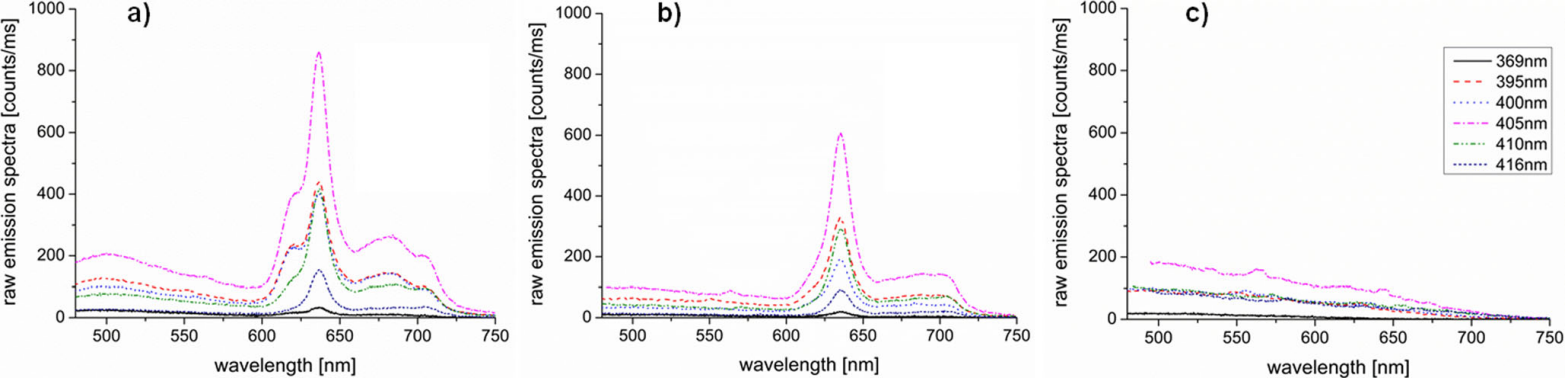
Typical emission spectra with subtracted dark spectrum at all six excitation wavelengths, 6 h after 5-ALA administration, of a biopsy specimen (dysplastic) on the CAM (**a**), the CAM adjacent to the biopsy specimen (**b**), and the CAM only (**c**)

Figure 3 shows typical emission spectra with subtracted dark spectrum before normalization at all six excitation wavelengths, 6 h after 5-ALA administration, of a biopsy specimen (dysplastic) on the CAM (a), the CAM adjacent to the biopsy specimen (b), and the CAM only (d). Fluorescence spectra of the biopsy specimens on the CAM showed PpIX fluorescence peaks at 636 nm. Maximum autofluorescence was observed around 500 nm. In contrast, the emission spectra adjacent to the biopsy specimens showed negligible autofluorescence and a clear PpIX fluorescence profile. The main difference between the biopsy specimens and the adjacent fluorescence was that the CAM did not show porphyrin fluorescence at the 620- and 680-nm emissions. Fluorescence spectra of the CAM only revealed slightly less autofluorescence compared to the biopsy specimen and negligible PpIX fluorescence compared to the biopsy specimens and the surrounding CAM.

The PpIX intensity ratios *I*_636_/*I*_600_ did not differ significantly between dysplastic and non-dysplastic tissue. Therefore, and due to the low amount of biopsy specimens classified as non-dysplastic, we decided to analyze all biopsy specimens (Table 2) per time point and excitation wavelength in one group, to which we refer further as BE tissue.

**Fig. 4:**
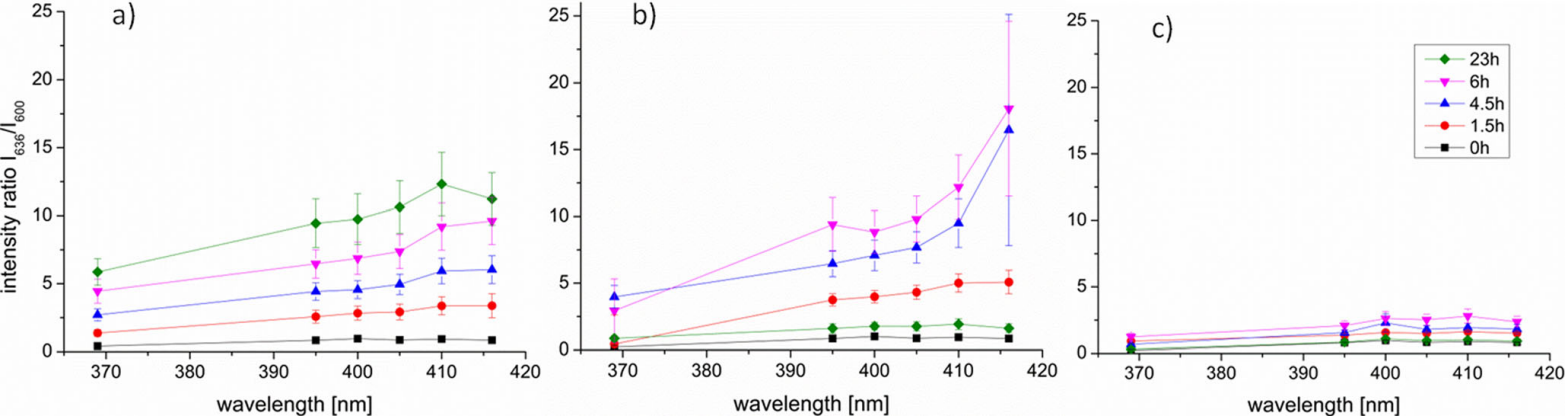
Mean PpIX intensity ratios *I*_636_/*I*_600_ with standard error of the mean at 0, 1.5, 4.5, 6, and 23 h after 5-ALA administration obtained from the BE tissue on the CAM (**a**), the CAM adjacent to the BE tissue (**b**), and the CAM only (**c**)

The intensity ratios *I*_636_/*I*_600_ of BE tissue showed increased PpIX fluorescence with increasing excitation wavelength and time point (Fig. 4a). Bonferroni’s multiple comparison tests of all pairs of time points showed a significant increase in the intensity ratios at 0 vs. 6 h, 0 vs. 23 h, 1.5 vs. 23 h, and 4.5 vs. 23 h at all excitation wavelengths. Intensity ratios at 369-nm excitation were significantly lower compared to 405-, 410-, and 416-nm excitation at all time points. The intensity ratios at 410- and 416-nm excitation at 4.5 h were significantly higher compared to 0 h. At 4.5 h after 5-ALA administration, the PpIX intensity ratios at 416-nm excitation were significantly higher compared to all other excitation wavelengths except 410 nm. Each measurement at 4.5 h after 5-ALA administration separately showed that in 96 % (23/24) of the cases, the highest PpIX fluorescence intensity ratios were obtained at 410-nm (63 %) and 416-nm (33 %) excitations. The lowest intensity ratios were obtained at 369-nm excitation in 96 % (23/24) of the cases. The intensity ratios at 410-nm excitation were 2.3 times higher (SD: ±0.8) compared to 369-nm excitation.

The intensity ratios *I*_636_/*I*_600_ on the CAM adjacent to the BE tissue (Fig. 4b) tend to increase faster and became higher compared to the BE tissue. There was a significant increase of the intensity ratios on the CAM adjacent to the BE tissue between time point 0 vs. 1.5 h, 0 vs. 4.5 h, 0 vs. 6 h, and a significant decrease at time points 4.5 vs. 23 h and 6 vs. 23 h at all excitation wavelengths except 369 nm. At 23 h after 5-ALA administration, the PpIX fluorescence was back at baseline level (*t* = 0). The PpIX fluorescence on the CAM only (Fig. 4c) increased significantly after 6 h, but those intensity ratios were negligible (five to seven times lower) compared to the intensity ratios obtained from the BE tissue or adjacent to it.

**Fig. 5:**
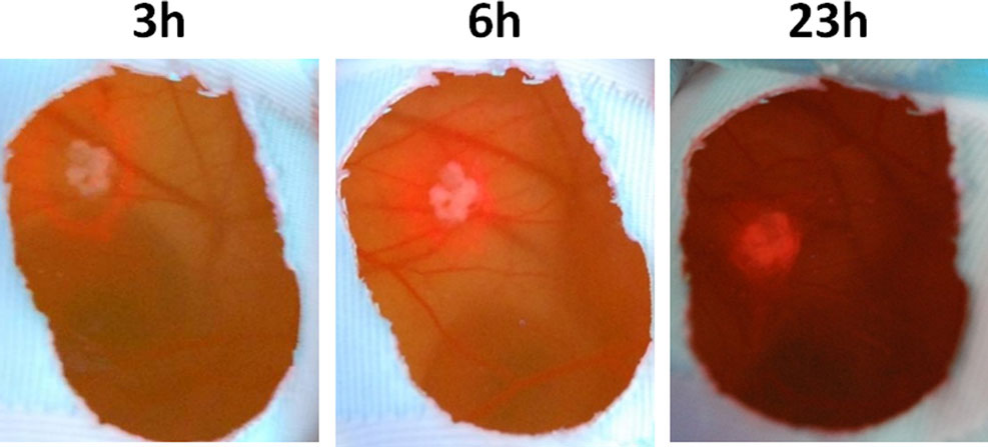
Fluorescence images of BE tissue on the CAM at 3, 6, and 23 h after 5-ALA administration

Fluorescence imaging was performed to obtain an overview of the PpIX distribution on the BE tissue and CAM. Fluorescence images (Fig. 5) taken several hours after 5-ALA administration showed first a strong red fluorescence surrounding the BE tissue and with increasing time increasing fluorescence of the BE tissue and decreasing fluorescence of the surrounding CAM.

## Discussion

The evaluation of the CAM model showed that biopsy specimens were still vital after 1.7 days on the CAM, on average. The lower intensities of the ex vivo autofluorescence spectra around 500 nm might be caused by the different measurement procedures; in vivo, the probe was in contact with the tissue site under investigation, whereas ex-vivo, the probe did not touch the biopsy specimen. Whereas, the higher intensities in the rest of the ex vivo autofluorescence spectra might be caused by lower blood absorption compared to the in vivo situation. Despite these deviations, the overall correlation of the ex vivo with the in vivo autofluorescence spectra showed good resemblance.

Previous research using the CAM model in combination with 5-ALA focused on tumor specimens and cell lines [21–24]. The observed conversion into PpIX in our study suggests the existence of a functional metabolism within the BE tissue. As expected, the lowest PpIX intensities were induced with the shortest wavelength, 369 nm, which is about 30 nm below the Soret band of PpIX. The highest PpIX intensities were induced at 410- and 416-nm excitations without a significant difference between them.

In our study, BE tissue showed increased PpIX fluorescence with increasing time, which is in agreement with known in vivo observations. The BE tissue showed no significantly reduced PpIX fluorescence after 23 h, which indicates a slower PpIX clearance compared to in vivo observations [25–28]. This increased clearance time might be caused by the topical bolus administration of ALA. The excessive ALA is not removed, and therefore, the tissue exposure is expected to be longer compared to in vivo conditions. Furthermore, the ALA diffusion into the tissue and redistribution within the egg might be prolonged in the CAM model. The in vivo kinetics were already studied [25–28]; therefore, we focused on studying the CAM model as a preclinical approach for fluorescence diagnostics with special interest in photosensitizer. This preclinical model benefits from no patient hazard, and it is a more physiological approach then studying cells in vitro. The fluorescence of the CAM adjacent to the BE tissue showed a faster increase and decrease of induced PpIX, whereas the PpIX fluorescence and autofluorescence of the CAM only showed negligible intensities. Fluorescence images showed that the high PpIX fluorescence adjacent to the biopsy specimens was due to the topical application of 5-ALA surrounding the biopsy specimens.

Although the CAM model was applicable for our research purpose, the spectroscopy measurements had several limitations. The spectroscopy set-up used a mounted probe, to ensure stable measurements. However, due to movements of the embryo and therefore repositioning of the BE tissue under the probe, equal distances and angles of the measurements cannot be assured and sometimes measurements needed to be repeated. The histological classification showed that around 40 % of the grafted biopsies did not stay vital on the CAM and therefore needed to be excluded from the analysis. This necessitates the usage of an increased amount of biopsies or an optimized protocol, for example, by leaving the biopsy specimens a maximum of 2 days on the CAM or daily administration of growth factors and/or medium to the tissue on the CAM which may prolong tissue vitality.

Differentiation between BE tissue and the CAM was possible, due to the differences in spectral shape, 5-ALA kinetics, and the wavelength-dependent intensity ratios. The main difference between BE tissue and the CAM spectra was that the CAM did not show porphyrin fluorescence at the 620- and 680-nm emissions (Fig. 3). This fluorescence might be caused by the formation of water-soluble porphyrins [29].

In conclusion, we showed that grafting freshly collected human BE biopsy specimens on the CAM is feasible. Our results suggest that the CAM model might be used to study fluorescence diagnostics such as autofluorescence and induced fluorescence behavior of human biopsy specimens on the CAM as a preclinical research tool. Further preclinical research is recommended, with an increased amount of biopsies from non-dysplastic and dysplastic BE tissue, to assess the potential in tissue discrimination. Furthermore, with the CAM model keeping human tissue vital for a few days, the responses to new photosensitizer might be evaluated. Besides that, our current fluorescence spectroscopy system can be modified by adding a white light source for additional reflectance spectroscopy which had shown its potential in endoscopic detection of dysplasia [30–32].
